# Supplementary material for: Development of a computer-aided design software for the quantitative evaluation of aesthetic damage
Source: PLoS One. 2019 Dec 18;14(12):e0226322. doi: 10.1371/journal.pone.0226322 (PMC6919621; doi:10.1371/journal.pone.0226322)
Supplement: S1 Code site — 1. (ZIP) [file pone.0226322.s001.zip › [EESC_jr][Projeto]Relatorio_07_21/reports/templates_zip/gentelella-master/documentation/index_cn.html]

Gentellela Alela! | 


进入主内容:


切换导航

Gentellela Alela! - 文档

- 关于
- 文件和目录
- 布局和结构
- 选项
- 信誉
- 更改 日志 v.1.9 -> v.1.9.2
- 更改 日志 v.1.8 -> v.1.9
- 联系方式

# 简介

欢迎到**Gentelella**,
一个响应式的，扁平的和全功能的管理模版，仅仅在ThemeForest上面有销售。这个模版基于Bootstrap 3,使用先进的技术例如HTML5和CSS3,可以用于任何规模的后端方案。

## 支持请求

支持浏览器包括 **IE9+, Google Chrome, Mozilla Firefox, Apple Safari, Opera 和所有Android和IOS移动浏览器**。对于那些不在列表中的浏览器，是不支持的。

## 文档范围

这份文档旨在解释 **结构像文件和目录，模版和JavaScript或者你可以配置的** 模版。 因为这份模版是建立在优秀的Bootstrap 3框架上，你可以在这里找到源文件。http://getbootstrap.com/css/

这份文档是假定你已经知道基本的 CSS,JavasScript和HTML,所有只有模版元素的使用和概念会覆盖在这里。

## 开始

开始，只需浏览一些示范页面。
使用 **Chrome Developer Tools (Chrome), Firebug (Firefox)**
或者相似的插件去分析代码。
为了能够更深入理解代码，打开HTML和JS文件，读注释的代码。

使用 `plain_page.html` 然后开始建立你自己的页面。

  
  

## 文件和目录

  
  

## 层次和结构

- 标题
- 顶层菜单
- 侧栏菜单
- 内容
- 页脚

#### 标题

下面是标题代码

```
        Gentellela Alela! |
```

#### 边栏菜单

导航菜单，展示个人简介和初级的功能例如登出，全屏和设置。

```
Gentellela Alela!


Welcome,

## Anthony Mutisya
```

#### 内容

标题和页面内容是被主要内容包裹着的

```
### Plain Page Page subtile


Go!


## Page title Page subtile


- - Settings 1
  - Settings 2
```

  
  

## 选项

Gentelella 提供了一些选择让你布局app.这里有：

- 固定的边栏
- 固定的顶部导航
- 固定的页脚

### 固定的边栏

缩小导航，因此用户可以有更多的空间在页面上，当你在父亲节点的图标上悬浮的时候。

  

### 固定的顶部导航

  
  

## 信誉和感谢

| 名称 | URL |
| --- | --- |
| jQuery | http://jquery.com/ |
| jQuery Sparkline | http://omnipotent.net/jquery.sparkline/ |
| Auto resize textarea | http://www.jacklmoore.com/autosize/ |
| Nice scroll | http://areaaperta.com/nicescroll/ |
| C3 charts | http://c3js.org/ |
| Full screen editor | http://burakson.github.io/fseditor/ |
| jQuery Tags Input | https://github.com/xoxco/jQuery-Tags-Input |
| jQuery Validation Plugin | http://parsleyjs.org/ |
| Colorpicker for Bootstrap | https://github.com/xaguilars/bootstrap-colorpicker |
| bootstrap-daterangepicker | https://github.com/dangrossman/bootstrap-daterangepicker |
| bootstrap-fileupload | http://blueimp.github.io/jQuery-File-Upload/ |
| bootstrap-multiselect | https://github.com/davidstutz/bootstrap-multiselect |
| bootstrap-wysihtml5 | http://jhollingworth.github.io/bootstrap-wysihtml5/ |
| Dropzone | http://www.dropzonejs.com/ |
| Bootstrap switch | https://github.com/nostalgiaz/bootstrap-switch |
| SwitcheryJs | http://abpetkov.github.io/switchery/ |
| DataTables | https://github.com/DataTables/DataTables |
| DataTables tools | https://github.com/DataTables/TableTools |
| easy pie chart | https://github.com/rendro/easy-pie-chart |
| FullCalendar | https://github.com/arshaw/fullcalendar |
| jQuery Knob | https://github.com/aterrien/jQuery-Knob |
| select2 | https://github.com/ivaynberg/select2/ |
| jQuery-Autocomplete | https://github.com/devbridge/jQuery-Autocomplete |
| Twitter Bootstrap Wizard | https://github.com/VinceG/twitter-bootstrap-wizard |
| E-Charts | http://echarts.baidu.com/doc/example-en.html |
| Font Awesome | http://fortawesome.github.io/Font-Awesome/ |
